# rna-tools.online: a Swiss army knife for RNA 3D structure modeling workflow

**DOI:** 10.1093/nar/gkac372

**Published:** 2022-05-17

**Authors:** Marcin Magnus

**Affiliations:** ReMedy International Research Agenda Unit, IMol Polish Academy of Sciences, Warsaw, Poland

## Abstract

Significant improvements have been made in the efficiency and accuracy of RNA 3D structure prediction methods in recent years; however, many tools developed in the field stay exclusive to only a few bioinformatic groups. To perform a complete RNA 3D structure modeling analysis as proposed by the RNA-Puzzles community, researchers must familiarize themselves with a quite complex set of tools. In order to facilitate the processing of RNA sequences and structures, we previously developed the rna-tools package. However, using rna-tools requires the installation of a mixture of libraries and tools, basic knowledge of the command line and the Python programming language. To provide an opportunity for the broader community of biologists to take advantage of the new developments in RNA structural biology, we developed rna-tools.online. The web server provides a user-friendly platform to perform many standard analyses required for the typical modeling workflow: 3D structure manipulation and editing, structure minimization, structure analysis, quality assessment, and comparison. rna-tools.online supports biologists to start benefiting from the maturing field of RNA 3D structural bioinformatics and can be used for educational purposes. The web server is available at https://rna-tools.online.

## Graphical Abstract

**Graphical Abstract:**
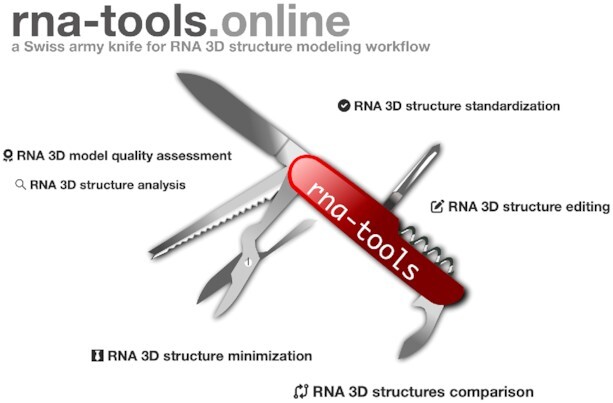
Overview of the tasks supported by rna-tools.online.

## INTRODUCTION

Ribonucleic acid or RNA is a central type of molecule in a broad variety of biological processes throughout the central dogma (1,2), RNA splicing (3), transcription regulation (4). The functional diversity of RNA is attributed to its ability to form specific 3D structures that can carry out biological functions (5). Thus, the accurate and rapid modeling of RNA 3D structure is an important step in understanding the molecular function as well as its interaction with other macromolecules and ligands.

During the last decade, a good number of RNA 3D structure prediction algorithms have been actively developed and improved. Improvements have been made in the efficiency and accuracy of RNA 3D structure prediction methods as reported in publications describing the RNA-Puzzles initiative (6–9). However, many tools developed in the field stay exclusive to only a few bioinformatic groups. To perform a complete RNA 3D structure modeling analysis as proposed in the RNA-Puzzles publications, researchers must familiarize themselves with a complex set of disparate tools, which often work at cross purposes. Several described tools are implemented in a form of a web server, such as methods for RNA 3D structure prediction: SimRNAweb (10), ROSIE/FARFAR2 (11,12), RNAComposer (13), however, many operations must be done on a local machine and are difficult to be applied without the knowledge of basic RNA bioinformatics.

In order to facilitate the processing of RNA sequences and structures, we previously developed the rna-tools package (14). The initial goal of the package was to provide a more abstract way to process data for RNA 3D modeling and analysis. Our work on the standardized dataset for the RNA-Puzzle community (14) made us realize how difficult it was to process structural files so they can be compared and the best models can be selected. A similar problem has been also recognized in the field of protein structure prediction and projects like pdb-tools (15) were developed. The focus of rna-tools was to (i) identify limitations of the existing tools (e.g. in the case of ModeRNA used here for introducing mutations, we developed a workaround so the program can process mutations in multiple chains), (ii) add advanced options to existing tools (e.g. for calculation of RMSD, advanced system of selection segments was introduced), (iii) identify the need for new tools (e.g. ‘diffpdb’ for text-based comparison of PDB files), (iv) integrate the existing tools and design ability to combine them into complex analyses. Nowadays, rna-tools has become a wide toolbox to approach every aspect of working with various types of RNA data. The package was used in various scientific projects, to calculate the stability of various U6 RNAs of the spliceosome (16), for years now to process input files obtained from users for SimRNAweb (10)(RNA 3D structure prediction method) and NPDock (17)(RNA/DNA protein docking method), and to analyze data for RNArchitecture (18) database (a classification system of RNA families with a focus on structural information) and EvoClustRNA (19) (RNA 3D structure prediction using multiple sequence alignment information). Moreover, the package was used to provide computational resources for the RNA-puzzle community (14) and also offered tools for biological applications developed by others, e.g. processing files for the development of a new scoring function for RNA-ligand interactions (20) and for an analysis of spliceosomal structures (21).

We realized that the next step for making RNA 3D structure modeling workflows even more accessible was to provide these tools online. Using rna-tools requires the installation of a mixture of libraries and programs, basic knowledge of the command line and the Python programming language. To open opportunities for the wider community of biologists to take advantage of the new developments in RNA structural biology, we developed the rna-tools.online. The web server provides a user-friendly platform to perform many standard analyses required for the typical modeling workflow: 3D structure manipulation and editing, structure minimization, structure analysis, comparison analysis, and quality assessment. There are tools that provide some of these functions. However, RNApolis (22) is a central hub that connects the user to a few independent tools that carry out specific tasks that are not directly integrated together. The ROSIE server (12) and the MolProbity server (23) offer only limited functionality for PDB file editing compared to the described server. The goal for rna-tools.online is to develop a more versatile platform with expert-selected tools to perform actions from every area of RNA 3D structure modeling and with a gentle learning curve for new users.

## MATERIALS AND METHODS

### Implementation

rna-tools.online and all utility programs were implemented in Python 3. All the tools implemented in the server leverage the use of the standalone rna-tools package, to which we added a way to input the data from the web (a page form for each tool) and a way to return the results of the computation and of the command line outputs. The web server was developed using the Django framework for the backend, while the frontend uses the jQuery JavaScript library to send and retrieve the data from the backend. Each tool collects the user inputs within a form. The uploaded files and the supplementary data are sent through an Ajax request to the backend when the user presses the ‘Run!’ button to start the computation. In the backend, the original rna-tools package is run on the server command line to perform the job requested by the user. The output of this job is then sent back through an Ajax response to the page the user is visualizing. At the end when the computation has processed the original files, the result files, a log file and a bash script are returned to the user as links. The user can download them by clicking on these links. The bash script can be used to re-run the tool on the user's local machine (if the local version of the rna-tools package is installed). The user will be able to later return to the job and its results by using a unique link that will be generated and displayed on the tool page at the end of the computation. Some tools use JSMol (24) in order to render a preview of their resulting 3D structures.

## RESULTS

**Figure 1.**
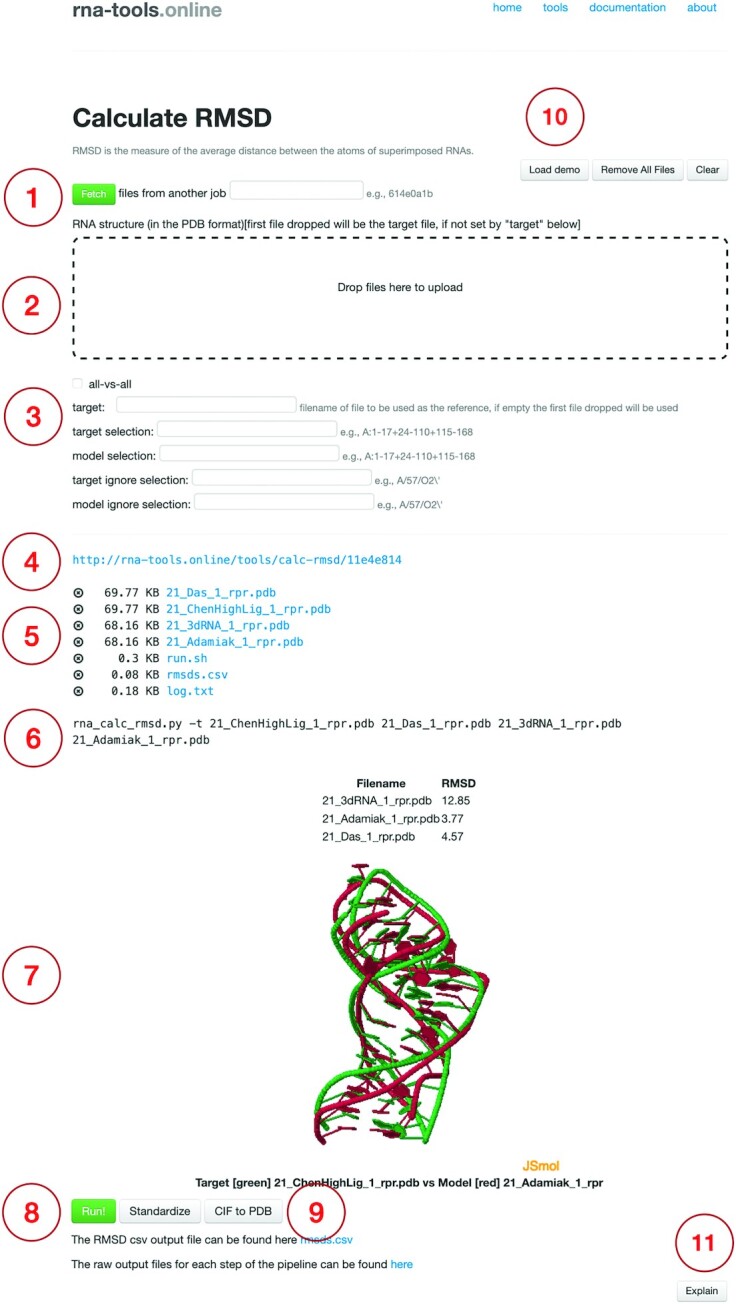
Example of a tool page ‘Calculate RMSD’. Analogous forms are provided for each tool. (1) Input files from other jobs can be fetched by specifying a unique job identifier (2) or can be uploaded by dropping files into the upload box. (3) Some tools may have specific options. For example, in ‘Calculate RMSD’, it is possible to calculate RMSD in an all-vs-all way or using different fragment selection options. (4) For each job a unique link is provided. This link can be saved, shared, or used to return the job results. The ending of this link is the unique job code that can be used in other tools of rna-tools.online to fetch its stored files. (5) The list of loaded files represents both, files uploaded by the user or generated by the tool. Each file can be downloaded or removed from the job folder. (6) To help the user understand how to run the same tool at the local machine, the executed command is displayed. (7) Results are presented in a tool-specific manner, here, a table of filenames and RMSD is displayed with a superposition rendered using JSmol for the reference structure and one of the models. (8) The main execution for a tool is triggered upon clicking ‘Run!’ (9) Auxiliary functionalities are also provided, in the case of this tool, the user can execute a standardization procedure of PDB files and conversion from the mmCIF file format to the PDB format before pressing ‘Run!’ in order to prepare the files. (10) A demo can be loaded in order to test the tool. (11) Furthermore, at the bottom of each form, a section with detailed documentation can be found.

The rna-tools.online server is designed to facilitate various procedures required in structural files analysis and preparation in the field of RNA 3D structural bioinformatics. The user can take advantage of multiple groups of tools that were divided into sections to help the user understand which tool to use for a given task. All tools on the web page follow a very similar pattern (Figure 1), are well documented and multiple examples are provided. By using a standard web browser, the user will be able to select a tool from a list of categorized rna-tools, follow the specific instructions given on the tool page to learn how to use it and what the tool is used for, upload the user's files and start a job execution. Once the computation is done, the web server allows the user to download the results and explore the command-line steps that were used to perform the analysis. This will also provide a way to learn how the rna-tool package can be used and will spur the user into trying to perform more customized analyses. For each job, a unique identifier is generated, this can be used to pass the output of one job to another job using the ‘Fetch’ functionality allowing users to create complex analyses by chaining together different tools. Below, we will provide a quick summary of tools available online.

### RNA 3D structure analysis

The first group of tools includes programs that aim to facilitate the analysis of RNA 3D structure. With ‘Get sequences’ the user can easily obtain RNA sequences for the uploaded PDB files. To obtain secondary structures from the PDB files the tool ‘Get secondary structures’ can be used based internally on 3DNA/DSSR (25) software. The 3DNA/DSSR software is also used for the next tool, ‘Analysis with X3DNA’ which provides various detailed statistics for PDB files such as a list of RNA elements (helixes, stems, motifs, nucleotide modifications) and configuration of base pairs. The last tool in this group uses ClaRNA (26) to classify the contacts (interactions) between base pairs in a PDB file.

### RNA 3D structure editing

The next group of tools allows the user to edit PDB files, most of them are using BioPython (27). The ‘Concatenate PDB files’ tool is able to merge two or more PDB files into one file. ‘Extract from PDB’ extracts and ‘Delete from PDB’ removes specified residues from a PDB file. ‘Edit PDB file’ edits specific residues in a PDB file. ‘Mutate residues in PDB file’ mutates residues in a PDB file using an improved (multiple chains) code with ModeRNA (28). ‘Swap chains name in PDB file’ swaps names of the chains in a PDB file, ‘Replace XYZ coordinates in PDB file’ replaces XYZ coordinates of one PDB file with XYZ coordinates from another file which is useful for homology modeling.

### RNA 3D structure standardization

This group of tools aims to facilitate operations on the standardization of RNA 3D structure. ‘Standardize PDB for RNA puzzle submission’ allows standardizing a PDB file to be compatible with the format proposed by the RNA-Puzzles community (14). The tool standardizes the naming of atoms, residues, and chains, reports and adds missing atoms removes water and ions, and keeps only canonical RNA atoms. ‘Standardize PDB for Molecular Dynamics’ allows standardizing a PDB file to be compatible with the format used in Molecular Dynamics, e.g. by OpenMM (29). The tool standardizes the naming of atoms, residues, and chains, reports and adds missing atoms removes water and ions, and keeps only canonical RNA atoms.

### RNA 3D structure minimization

A common task in RNA bioinformatics is to remove steric clashes and optimize bonds and angles of an RNA model. We provide two tools: ‘Minimize with QRNAS’ which offers structural minimization based on QRNAS (30), a software tool for fine-grained refinement of nucleic acid structures, and ‘Minimize with OpenMM’ which offers another type of structural minimization for PDB files based on OpenMM (29), a molecular dynamics simulation toolkit.

### RNA 3D structures comparison

The next group contains three tools that can be used to compare structural files. The ‘diffpdb’ tool is a simple program to perform a text-based comparison of two files of PDB format to identify the difference in the annotation of atoms, missing atoms, and missing fragments. ‘Calculate Root Mean Square Deviation (RMSD)’ can be used to calculate an RMSD (31) that is a measure of the average distance between the atoms of superimposed RNAs. The tool at the server provides options for the selection of fragments in the target structure and structures used for comparison, as well as the exclusion of specific atoms (Figure 1). RMSD is a relatively simple, geometrical measure, useful in some scenarios, however for RNA comparison more useful can be a measurement that takes into account interactions networks of RNA molecules. We provide ‘Calculate Network Interaction Fidelity (INF)’ where RNAs are represented by a network of interactions, and the closer two networks of interactions of two molecules are similar, the higher the INF score (32).

### RNA 3D model quality assessment

The last step of RNA 3D structure modeling is the assessment of the quality of a model. The web server provides two tools that are executed to obtain predicted quality scores: RASP (33) and Dfire (34). In both cases, lower scores mean a higher probability of a given structural model being of good quality.

### Conversion between mmCIF and PDB

As only a limited number of chains and atoms can be deposited in the PDB format, the mmCIF format (35,36) has been introduced to provide an alternative way to save structures. As the predicted RNA structures are normally within the capability of the PDB format, this format is still used in the RNA-Puzzles community. Moreover, many tools available in the field of RNA 3D bioinformatics still are using the PDB format and likely will not be updated for the mmCIF format. Thus, we decided to provide a web application to convert mmCIF format to PDB format (‘Convert CIF files to PDB’) and reverse (‘Convert PDB files to CIF’). These two tools are based on the open-source version of PyMOL (37).

## DISCUSSION

The rna-tools.online server offers a new user-friendly interface for the rna-tools package, delivering RNA 3D structure modeling tools to any user. The server accepts RNA structural files and enables various analyses. On the documentation page (https://rna-tools.online/docs), the user can find a detailed description of how the tools can be used and how to interpret the results. All tools are well documented and examples are provided to help the users to understand the tools. Advanced applications are possible after downloading output scripts and files to a local machine. The rna-tools website and its stand-alone version are rigorously maintained and we are open to providing additional tools and addressing requests from the general community. Moreover, we plan also to enhance the infrastructure to provide more computational power, especially for molecular dynamics-based functions. We hope that the user will be encouraged by the possibility to replicate the execution of these tools on their local machine to perform even more complex analyses using the tools provided by the rna-tools stand-alone version or similar packages, e.g. Barnaba (38). We encourage users to report any problems and requests using the Issues system of the GitHub web page or to send feedback directly. We believe that our user-friendly server will encourage biologists to start benefiting from the maturing field of RNA 3D structural bioinformatics and that rna-tools.online will prove to be an important tool in both research and educational contexts.

## DATA AVAILABILITY

The web server is available at https://rna-tools.online. This website is free and open to all users and there is no login requirement.
